# Beta-blockers and glioma: a systematic review of preclinical studies and clinical results

**DOI:** 10.1007/s10143-020-01277-4

**Published:** 2020-03-14

**Authors:** Ishaan Ashwini Tewarie, Joeky T. Senders, Alexander F. C. Hulsbergen, Stijn Kremer, Marike L. D. Broekman

**Affiliations:** 1 Department of Neurosurgery, Haaglanden Medical Center, The Hague, The Netherlands; 2 Faculty of Medicine, Erasmus University Rotterdam/Erasmus Medical Center, Rotterdam, The Netherlands; 3 Computational Neurosciences Outcomes Center, Department of Neurosurgery, Brigham and Women’s Hospital, Harvard Medical School, Boston, MA USA; 4 Department of Neurosurgery, Leiden University Medical Center, Leiden, The Netherlands

**Keywords:** Beta-blockers, Neurosurgery, Glioma, Overall survival

## Abstract

Given the median survival of 15 months after diagnosis, novel treatment strategies are needed for glioblastoma. Beta-blockers have been demonstrated to inhibit angiogenesis and tumor cell proliferation in various cancer types. The aim of this study was to systematically review the evidence on the effect of beta-blockers on glioma growth. A systematic literature search was performed in the PubMed, Embase, Google Scholar, Web of Science, and Cochrane Central to identify all relevant studies. Preclinical studies concerning the pharmacodynamic effects of beta-blockers on glioma growth and proliferation were included, as well as clinical studies that studied the effect of beta-blockers on patient outcomes according to PRISMA guidelines. Among the 980 citations, 10 preclinical studies and 1 clinical study were included after title/abstract and full-text screening. The following potential mechanisms were identified: reduction of glioma cell proliferation (*n* = 9), decrease of glioma cell migration (*n* = 2), increase of drug sensitivity (*n* = 1), induction of glioma cell death (*n* = 1). Beta-blockers affect glioma proliferation by inducing a brief reduction of cAMP and a temporary cell cycle arrest in vitro*.* Contrasting results were observed concerning glioma cell migration. The identified clinical study did not find an association between beta-blockers and survival in glioma patients. Although preclinical studies provide scarce evidence for the use of beta-blockers in glioma, they identified potential pathways for targeting glioma. Future studies are needed to clarify the effect of beta-blockers on clinical endpoints including survival outcomes in glioma patients to scrutinize the value of beta-blockers in glioma care.

**Electronic supplementary material:**

The online version of this article (10.1007/s10143-020-01277-4) contains supplementary material, which is available to authorized users.

## Introduction

Glioblastomas are the most common primary malignant brain tumors [1]. With a median survival of just 16 months despite neurosurgical resection and chemoradiation, prognosis is poor and novel treatment strategies are needed [2–6].

Beta-blockers are competitive antagonists of the sympathetic effects of catecholamines on beta-adrenergic receptors [7, 8]. They have affinity for beta-1-, beta-2-, or beta-3-adrenergic receptors but do not evoke a response from these receptors. Therefore, the binding of a beta-blocker and adrenergic receptor prevents the binding and subsequent effects of catecholamines [9]. In cardiovascular diseases, beta-blockers are widely prescribed to counter these effects, including an increase in heart rate and blood pressure. Within the field of oncology, catecholamines have been demonstrated to stimulate secretion of pro-angiogenic factors such as vascular endothelial growth factor (VEGF) and boost tumor migration in several cancer cell lines [10–12]. Consequently, beta-blockers have been reported to inhibit angiogenesis and tumor cell proliferation in breast cancer, multiple myeloma, pancreatic cancer, and neuroblastoma cell lines by decreasing catecholamine-driven proliferation [13–16]. While the presence of beta-1-, beta-2-, and beta-3-adrenergic receptors has been demonstrated in glioma cell lines [17–19], the potential efficacy of beta-blockers in this cancer type remains to be elucidated [20]. The aim of this study was to systematically review preclinical and clinical studies on the effects of beta-blockers on glioma.

## Methods

### Search strategy

A systematic literature review according to the Preferred Reporting Items for Systematic Reviews and Meta-Analyses (PRISMA) guidelines [21]. The Embase, Medline Ovid (PubMed), Web of Science, Cochrane Central, and Google Scholar databases were searched to identify all relevant articles up to May 10, 2019. A professional librarian was consulted in order to construct a search syntax, which used synonyms for glioma and beta-blockers (suppl. Table 1). All selective and non-selective beta-blockers were included in the syntax. Clinical studies were included if they investigated the association between beta-blocker use and survival or other outcomes in glioma patients. Furthermore, all preclinical studies that investigated the effect of beta-blockers in glioma cell lines or in animal models were included as well. Case reports or articles written in languages other than Dutch or English were excluded. No restrictions based on the date of publication were used. This systematic search was complemented by screening the references of the included articles to identify additional publications. Titles and abstracts of retrieved articles were first screened by two independent authors (IRT, SK). Afterwards, two authors read the full text of potentially suitable articles separately (IRT, SK). Discrepancies were resolved by discussion and, if necessary, a third reviewer was consulted (JS).

### Data extraction

The following data were extracted from the included preclinical studies: year of publication, name of the first author, type of cell line, beta-blocker used, optimum concentration, cyclic adenosine monophosphate (cAMP) formation, morphology, and percentage of beta-adrenergic receptor blocked as determined relative to all cells on the plate. From clinical studies, the following parameters were extracted: year of publication, name of the first author, glioma categorized by grade, beta-blocker used, number of patients, 1-year survival rate, and median overall survival in months.

## Results

**Fig. 1:**
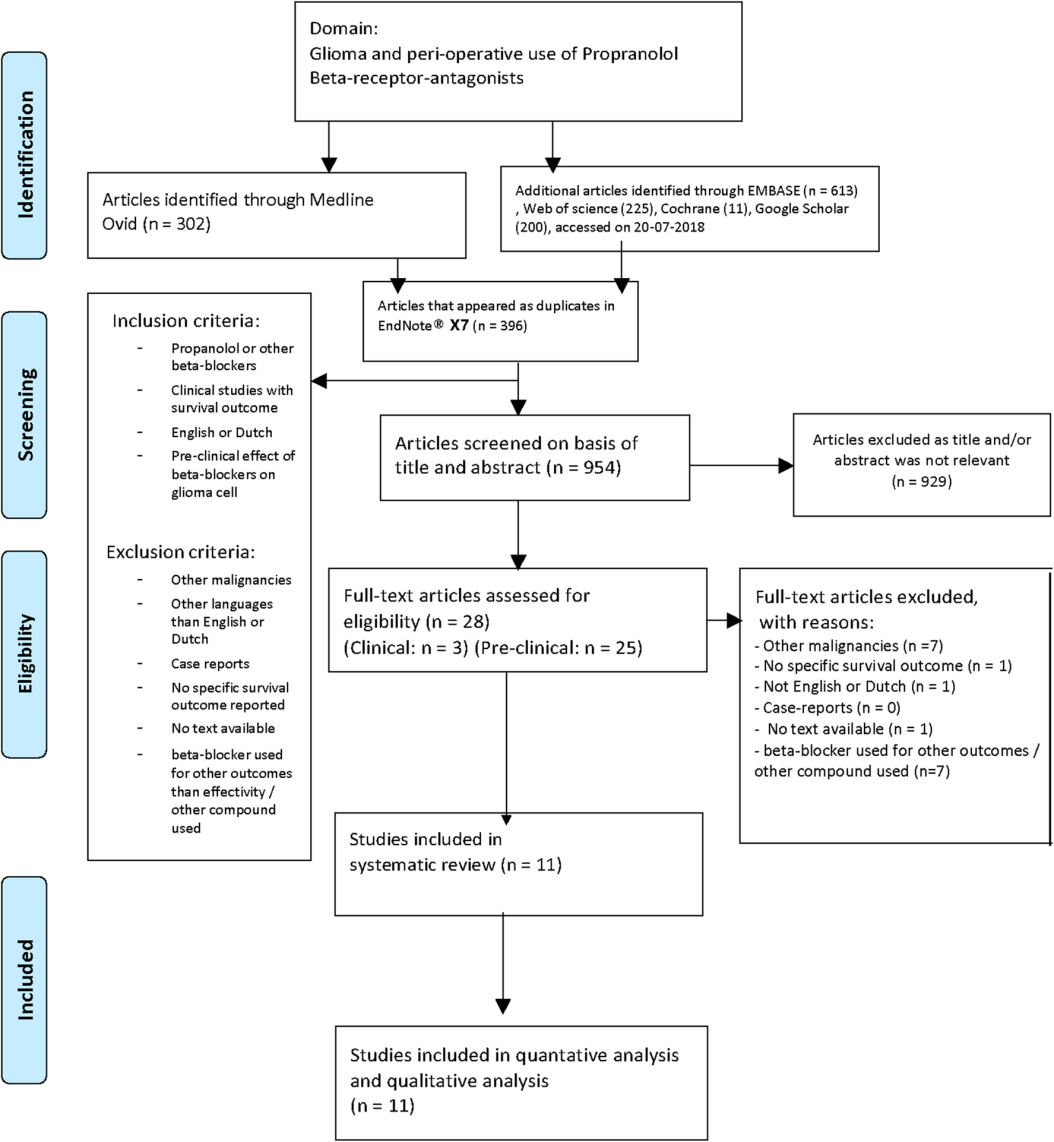
Flowchart

The search identified 980 unique studies. After screening of title and abstract followed by screening of the full text, we included 11 studies (10 preclinical and one clinical) that examined the effect of beta-blockers on glioma growth and patient outcomes (Fig. 1) [18, 20, 22–30]. Characteristics of all included preclinical studies can be found in Table 1.

**Table 1:** Characteristics of preclinical articles

Author	Year	β-Blocker	Cell line	β-Agonist	Anti-tumor effect	Main observation	*Δ* control group*
Edström et al.	1975	Sotalol	Human glioma cells	Isoproterenol	Antiproliferative	cAMP decrease	92%
Terasaki et al.	1979	Propranolol	Rat C6 glioma cells	Isoproterenol	Antiproliferative	cAMP decrease	x
Homburger et al.	1984	Pindolol	Rat C6 glioma cells	Isoproterenol	x	< 100% β-receptors blocked	52.2%
Homburger et al.	1985	Pindolol	Rat C6 glioma cells	Isoproterenol	x	< 100% β-receptors blocked	85%
Balmforth et al.	1986	Propranolol	G-CCM^a^	Isoprenaline	Inhibition secondary sites	x	x
Conroy et al.	1988	Propranolol	AC glioma cells	Isoproterenol	Antiproliferative	Decrease in stellate morphology	45%
Sokolowska et al.	2005	Propranolol	Rat C6 glioma cells	Isoprenaline	Inverse agonism	cAMP decrease	80%
Annabi et al.	2009	Propranolol	HBMEC^b^	x	Anti-angiogenic	MMP-9 decrease	40%
Erguven et al.	2009	Carvedilol	Rat C6 glioma cells	x	Antiproliferative	cAMP decrease	80%
Pavlova et al.	2018	Propranolol	In vivo	x	Antimigration	Decrease in migration/increase in survival	20%/50%

The "x" implies that they have not reported or did not use this variable in the study

^a^*G-CCM* human astrocytoma cell line

^b^HBMEC human microvascular endothelial cells of the brain

*All shown percentages demonstrate the relative difference in the main observation between control group (beta-agonist + cell line) and beta-blocker + beta-agonist + cell line

The identified preclinical studies investigated the following mechanisms through which beta-blockers could affect glioma growth: (i) reduction of glioma cell proliferation [18, 20, 23–26, 28, 29], (ii) decrease of tumorigenesis and glioma cell migration [22, 27], (iii) increase of drug sensitivity [24], and (iv) induction of cell death [24].

### Reduction of glioma cell proliferation

Eight studies investigated the effect of beta-blockers on glioma cell proliferation [18, 20, 23–26, 28, 29]. Two pathways were identified through which beta-blockers could potentially reduce glioma cell proliferation: a decrease in intracellular cyclic adenosine monophosphate (cAMP) levels resulting in lower cell activity [18, 20, 23–26, 28, 29] and a time-dependent cell cycle arrest [18].

### cAMP and cell metabolism

**Fig. 2:**
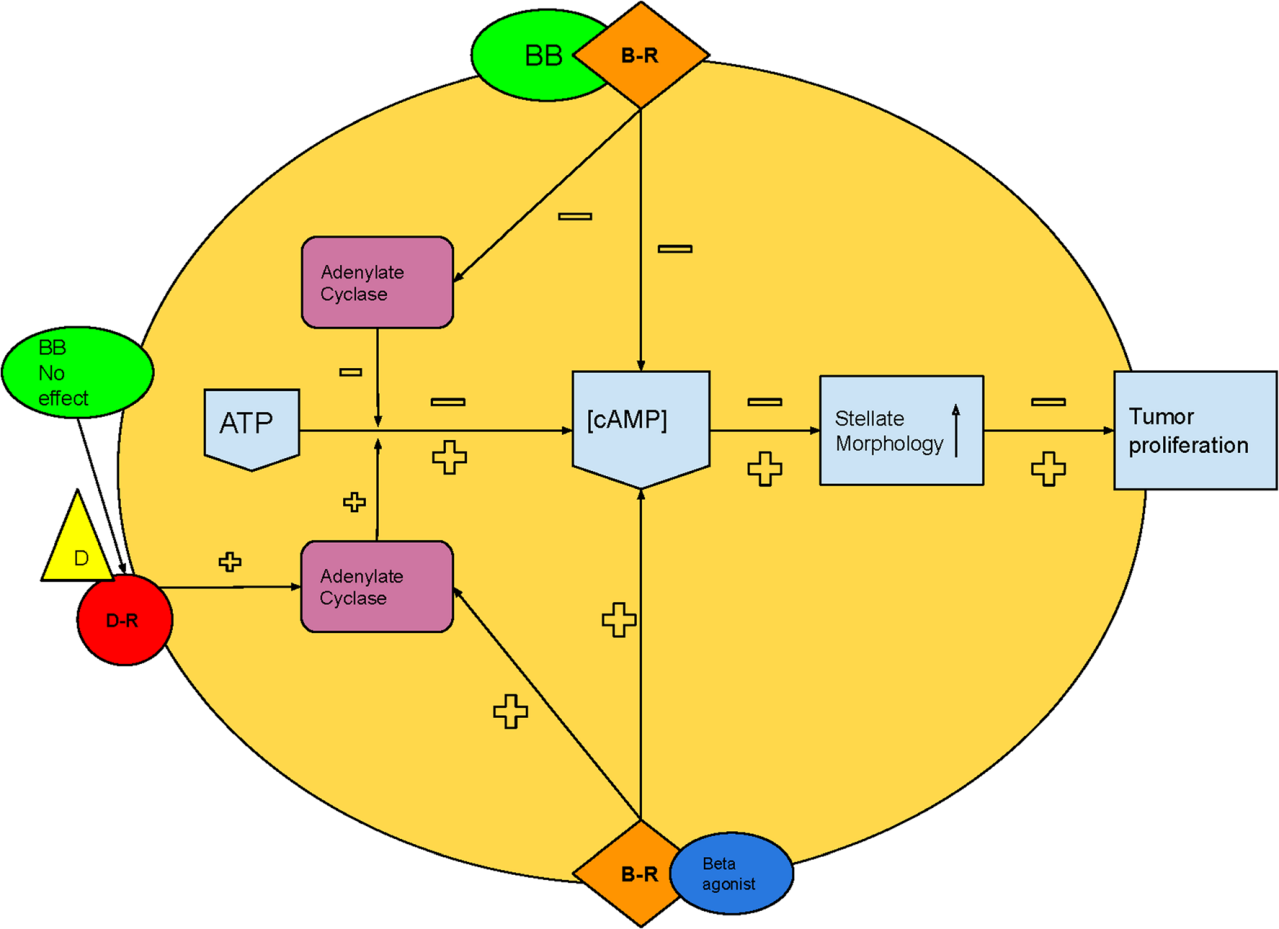
Schematic illustration of cAMP pathway

In two in vitro studies, elevated cAMP levels were associated with increased glioma cell proliferation and stellate transformation [18, 24]. Stellate transformation indicates that the (tumor) cell is more active, i.e., cytoplasmatic processes rise. Therefore, overall cell activity and proliferation are stimulated in this formation. This is a negative development in the treatment of a tumor. The spherical morphology could be induced by beta-agonists and suppressed by beta-blockers [18]. This process indicates increased tumor cell activity and extensive proliferation and could be induced by beta-agonists and suppressed by beta-blockers [18]. A schematic representation of the cAMP pathway in glioma can be found in Fig. 2. Six studies further investigated the relationship between beta-blockers and reduced cAMP levels, as well as the underlying pathways [23–26, 28, 29]. First, beta-blockers initiated a time- and dose-dependent decrease in cAMP formation caused by a blockade of the substrate (i.e., beta-agonists) bound activity [18, 24–26, 28, 29]. Second, suppression of the unbound, constitutive activity of adrenergic receptors was observed (i.e., beta-blockers functioning as inverse agonists) [28]. In three studies, however, maximum levels of cAMP formation could still be reached despite a blockade of > 90% of the beta-adrenergic receptors. This indicates that cAMP formation possibly occurs at receptor sites other than the beta-adrenergic variant [23, 28, 29]. Lastly, beta-blockers initiated a time- and dose-dependent decrease in adenylate cyclase, a protein that catalyzes the conversion of adenosine 5-triphosphate (ATP) to cAMP [18, 23–26, 28, 29].

### Time-dependent cell cycle arrest

Erguven et al. explored the effect of beta-blockers on the cell cycle [24]. In their study, carvedilol appeared to increase the percentage of glioma cells in the mitotic S- and G2-phases at 24 h after administration. However, it induced a cell cycle arrest in the G0/G1 phase at 72 h after administration.

### Decrease of glioma cell migration

Two studies examined the effect of beta-blockers on angiogenesis and glioma cell migration [22, 27]. Pavlova et al. injected rats with rat C6 glioma cells and treated half of them with ICI-118551 p.o., a specific beta-2-receptor antagonist, starting from the first day after implantation [27]. Upon implantation of the C6 glioma cells into the caudate putamen area, the control group was divided in rats that received either saline or isoproterenol in the same volume. The rats that received ICI-118551 survived longer than the control group (median 45 versus 20 days). All rats that received isoproterenol developed metastasis in the first 7 days after implantation. Furthermore, confocal imaging of fluorescent gliomas in vivo and ex vivo revealed that blockade of the beta-2-adrenergic receptors decreased glioma cell migration by 20% and reduced the disruption of the blood-brain barrier significantly (*p* < 0.001).

Annabi et al. did not observe a direct association with cell migration in vitro but reported an association between propranolol administration and matrix metalloproteinase nine (MMP-9) and Hu antigen R (HuR) [22]. MMP-2 was not affected by propranolol. MMP-9 and MMP-2 are associated with the integrity of the blood-brain barrier [31, 32] and have been shown to increase the invasiveness of human glioma cells [33, 34]. Annabi et al. observed that MMP-9 levels were positively correlated with the expression of beta-adrenergic receptors and reduced after treatment with propranolol [22]. Moreover, capillary structure formations were inhibited by propranolol in vitro. HuR and RNA-binding proteins are known to positively affect proliferation, survival, and translational efficiency in other cancer types including lung, breast, ovarian, and colon cancer cells [35–41]. Annabi et al. observed that propranolol does not directly affect HuR expression, but that it inhibits HuR translocation into cytosol where it stabilizes MMP-9 [22].

### Increase of drug sensitivity

One study investigated the effect of beta-blockers on the sensitivity of glioma cells to cytotoxic drugs [24]. In this in vitro study, Erguven et al. targeted the p-glycoprotein (p-Gp), a key efflux transporter in the blood-brain barrier known to attenuate the efficacy of various drugs by limiting transport to the central nervous system [42, 43]. Carvedilol administration in combination with imatinib, a kinase inhibitor that is primarily used for leukemia, resulted in increased imatinib-induced cell death [24].

### Induction of glioma cell death

**Fig. 3:**
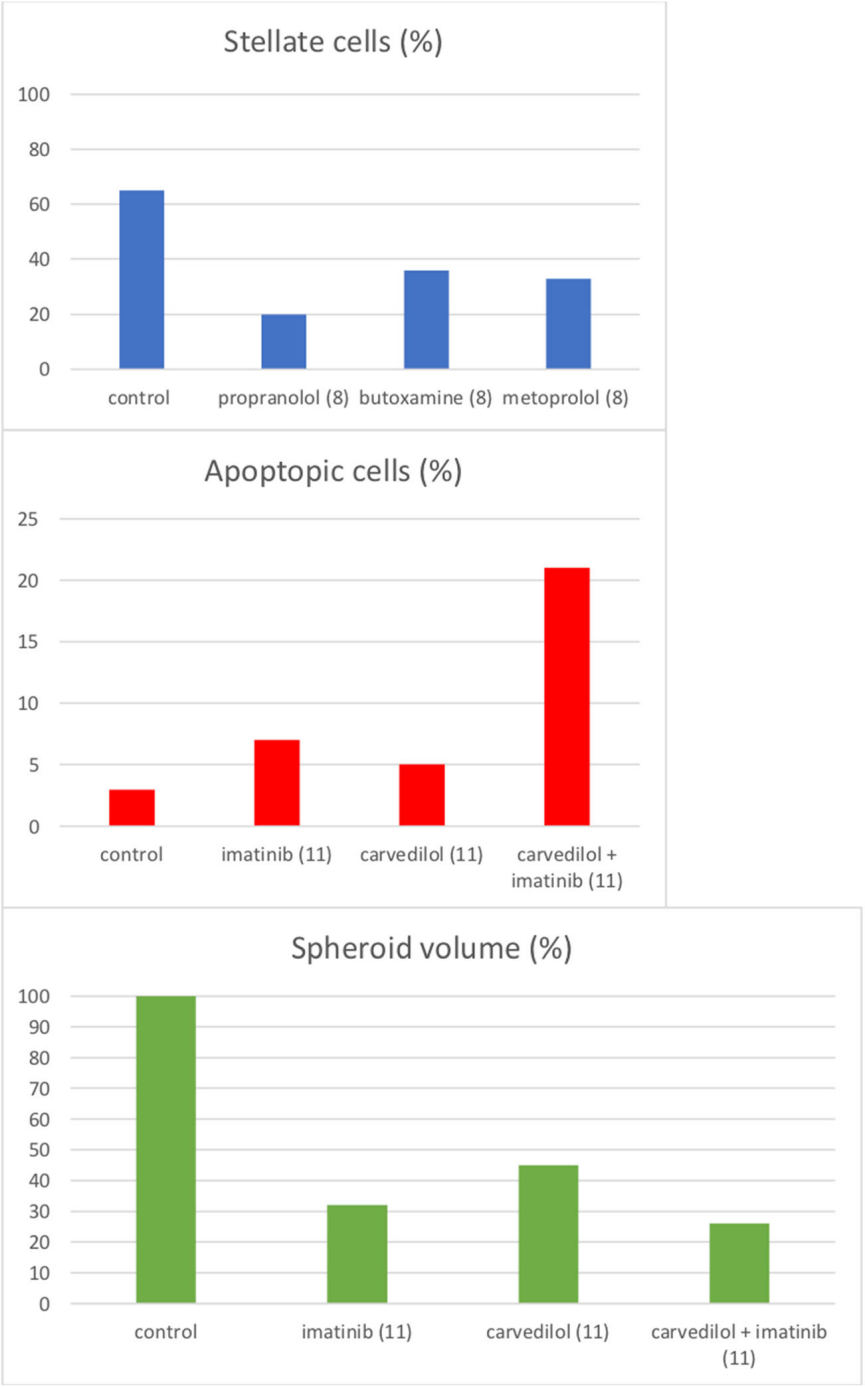
Morphological observations and effect on glioma cell proliferation

Erguven et al. also examined the effect of beta-blockers on programmed cell death [24]. They showed that carvedilol treatment established lytic changes in glioma cells, especially in the mitochondria, thereby initiating cell apoptosis. A single dose of carvedilol induced apoptotic cell death of 5% in all monolayer-cultured C6 glioma cells after 24 h (*p* < 0.05). At 72 h, this percentage of apoptotic cells was decreased to 2% (*p* < 0.01) [24]. The underlying mechanism, however, remains to be elucidated [24] (Fig. 3).

### Clinical evidence of beta-blockers affecting glioma

Johansen et al. studied the effect of beta-blockers on recurrent glioblastoma in a retrospective cohort of 218 patients [30]. This study compared patients that received beta-blockers with patients that did not receive any antihypertensive drug, while both groups were being treated with bevacizumab. After adjusting for multifocal disease, use of steroids, WHO performance status, and neurocognitive deficits, no association was found between beta-blocker usage and overall survival (hazard ratio 0.79; CI 95% 0.38–1.65; *p* value 0.53) or progression-free survival (hazard ratio 0.78; CI 95% 0.36–1.68; *p* value 0.52).

## Discussion

This systematic review synthesizes all preclinical and clinical evidence on the effect of beta-blockers on glioma growth and patient outcomes. Preclinical studies have identified several potential mechanisms of action through which beta-blockers might have a potential effect on (i) glioma cell proliferation [18, 20, 23–26, 28, 29], (ii) glioma cell migration [22, 27], (iii) drug sensitivity [24], and (iv) glioma cell death [24]. First, beta-blockers were demonstrated to lower tumor cell proliferation by decreasing levels of cAMP with dose and time dependency. Second, after administering beta-blockers a time-dependent cell cycle arrest was observed [18, 20, 23–26, 28, 29]. Third, blockade of the beta-2-adrenergic receptor decreased glioma cell migration [27]. Last, p-glycoprotein expression is possibly altered by beta-blockers resulting in an increase in drug sensitivity. One observational clinical study was included, which did not find an effect of beta-blockers on overall survival or progression-free survival in recurrent glioblastoma [30].

Beta-blockers have been suggested to reduce the risk of prostate cancer [44], as well as hepatocellular carcinomas in hepatitis C patients [45], and to prolong survival and reduce mortality in breast cancer patients [46]. In addition, it has been demonstrated that beta-blockers affect cell proliferation and migration in neuroblastoma cell lines [13]. Moreover, beta-blockers were demonstrated to elevate the therapeutic concentration of co-administered medications. Interestingly, this elevation of drug sensitivity did not seem to be related to a variation in p-Gp expression [13].

The current literature provides limited evidence to integrate beta-blockers into glioma treatment. However, the preclinical studies have identified various mechanisms through which the application of non-selective beta-blockers could potentially attenuate glioma cell proliferation.

Microglia have been demonstrated to upregulate matrix-altering enzymes, especially MMP-2 and MMP-9, to facilitate glioma infiltration. Downregulation of the MMP-cascade results in less glioma invasion, angiogenesis, and growth [47]. This systematic review identified contrasting results concerning the effect of propranolol on cell migration and the potential role of MMP-2 and MMP-9 [22, 27]: MMP-2 levels were not affected by propranolol, while MMP-9 levels were. Still, propranolol was not observed to directly affect glioma cell migration. This could indicate that even though both MMP-2 and MMP-9 affect migration, blocking one of these does not preclude alternative pathways from promoting cell migration. Although MMP-9 and MMP-2 are mostly similar substrates, it is hypothesized that they are regulated by different mediators, expressed under different conditions, and therefore have different instances in which they affect the tumor micro-environment [48, 49]. However, more studies are needed to elucidate how MMP-9 and/or MMP-2 directly or indirectly affect glioma cell migration in patients.

Finally, the observational study did not find a significant effect of beta-blockers on the survival rate of recurrent glioblastoma patients. In the multivariate analysis, neurocognitive deficits and steroids were included without reported association with overall survival. The aforementioned variables were included as a result of a study which reviewed variables influencing the quality of life and prognosis in glioblastoma patients [50]. The statistical insignificance of the hazard ratio of 0.79 could be due to an underpowered analysis. Moreover, the research question of this study was focused on angiotensin system inhibitors and the sample size of the sub-analysis regarding beta-blockers was not reported [30]. Pavlova et al. [27] observed a survival increase in rats with glioma. The significant decrease of glioma cell migration combined with the observation that all rats receiving isoproterenol developed metastasis within the first 7 days after implantation emphasizes the possibility of the beta-adrenergic pathway being a tumor migration pathway [27]. However, additional studies are still needed to elucidate this question.

There are limitations to the present review. Most studies predominantly focused on associations between beta-blockers and mediator enzymes (such as MMP-9) instead of examining their direct effect on clinical (e.g., survival and tumor progression) or physiological endpoints (e.g., cell proliferation and cell migration). As such, no inferences can be drawn on the direct association between beta-blockers and these endpoints*.* Studies that consider mediating proteins as surrogate endpoints rely on the strong assumption that these mediators are included in the causal pathway. Outcomes such as cell growth or migration are independent of such assumptions and thus would be able to support more robust conclusions. Additionally, this systematic review is subject to publication bias. Despite these limitations, we believe this review provides valuable insights into the potential utility of beta-blockers in glioma care and valuable target mechanisms for the development of anti-glioma drugs. To the best of our knowledge, this is the first systematic review synthesizing the current body of evidence on the effects of beta-blockers on glioma growth and patient outcomes, both in the preclinical and clinical realms.

## Future research

Preclinically, almost all studies were performed in vitro. Therefore, future research on the effect of beta-blockers in addition to conventional treatment of glioma should ideally be performed in vivo. Preclinical studies could further investigate the effect of beta-blockers in vivo by measuring clinically relevant outcomes (e.g., survival and recurrence rates) and physiological endpoints (e.g., both cell proliferation and cell migration) in mice. Preliminary results of an ongoing clinical study suggest that propranolol in combination with etodolac (VT-122) might have a positive effect on survival in recurrent glioblastoma patients. Hypothetically, VT-122 attenuates inflammation and thereby increases tolerability for anticancer therapy [51, 52]. Additionally, including medication and beta-blocker usage in large prospective neuro-oncological registries could be valuable in investigating the effect on cancer-related patient outcomes.

## Conclusion

Although preclinical research provides limited evidence for the effectiveness of beta-blocker usage in glioma care, this review identifies potential mechanisms through which beta-blockers might affect glioma proliferation, migration, drug sensitivity, and programmed cell death. However, the effect on patient outcomes remains unclear due to the limited body of clinical evidence. In addition to identifying potential mediators, future preclinical research should further explore the effect of beta-blockers on physiological and clinical endpoints. Including medication and beta-blocker usage in large prospective neuro-oncological registries could be a valuable step for examining the direct or conjunct effect on cancer-related patient outcomes.

## Electronic supplementary material

ESM 1 (PDF 221 kb).
