# Supplementary material for: Beta-blockers and glioma: a systematic review of preclinical studies and clinical results
Source: Neurosurg Rev. 2020 Mar 14;44(2):669–77. doi: 10.1007/s10143-020-01277-4 (PMC8035104; doi:10.1007/s10143-020-01277-4)
Supplement: Supplementary file 1 — (PDF 221 kb). [file 10143_2020_1277_MOESM1_ESM.pdf]

Suppl1. Search syntax in different databases

**embase.com 613**
('glioma'/exp OR 'brain cancer'/de OR 'central nervous system cancer'/de OR 'central nervous system tumor'/de OR 'brain tumor'/de OR (glioma* OR astrocytom* OR xanthoastrocytom* OR gliosarcom* OR oligodendrogliom* OR ependymom* OR glioblastom* OR ((brain OR 'central nervous system' OR cns OR intracerebral* OR cerebral* OR glial) NEAR/3 (cancer* OR cavernoma* OR neoplas* OR tumo* OR malign*))):ab,ti) AND ('beta adrenergic receptor blocking agent'/exp OR (((beta OR β) NEAR/3 (block* OR antagonis* OR sympatholytic* OR adrenolytic*)) OR adaprolol* OR afurolol* OR alprenolol* OR befunolol* OR bfe-55* OR bopindolol* OR bornaprolol* OR bromoacetylalprenololmenthane* OR bucindolol* OR bucumolol* OR bufetolol* OR bufuralol* OR bunitrolol* OR bunolol* OR bupranolol* OR butofilolol* OR carazolol* OR carpindolol* OR carteolol* OR carvedilol* OR cloranolol* OR deacetylmetipranolol* OR dexpropranolol* OR diacetolol* OR dichlorisoprenaline* OR dihydroalprenolol* OR dilevalol* OR diprafenone* OR ersentilide* OR exaprolol* OR falintolol* OR falintolol-oxalate* OR flestolol* OR hydroxybenzylpindolol* OR indenolol* OR iodopindolol* OR iprocrolol* OR isamoltane* OR isoxaprolol* OR labetalol* OR levobunolol* OR levomoprolol* OR mepindolol* OR mercuderamide* OR metipranolol* OR moprolol* OR nadolol* OR nifenalol* OR oberadilol* OR oxprenolol* OR pafenolol* OR pamatolol* OR penbutolol* OR pindolol* OR primidolol* OR prizidilol* OR procinolol* OR pronetalol* OR propranolol* OR proxodolol* OR ridazolol* OR soquinolol* OR sotalol* OR spirendolol* OR tazolol* OR tertatolol* OR tienoxolol* OR tilisolol* OR timolol* OR tolamolol* OR toliprolol* OR trasitensin* OR trepress* OR tribendilol* OR viskaldix* OR xibenolol* OR zoleprodolol* OR acebutolol* OR arotinolol* OR atenolol* OR bendacalol* OR bendacalol* OR betaxolol* OR bevantolol* OR bfe-55* OR bisoprolol* OR bopindolol* OR bornaprolol* OR bromoacetylalprenololmenthane* OR bucindolol* OR bucumolol* OR bufetolol* OR bufuralol* OR bunitrolol* OR bunolol* OR bupranolol* OR butofilolol* OR butoxamine* OR carazolol* OR carpindolol* OR carteolol* OR carvedilol* OR celiprolol* OR cetamolol* OR cicloprolol* OR cloranolol* OR cyanoiodopindolol* OR cyanopindolol* OR deacetylmetipranolol* OR dexpropranolol* OR diacetolol* OR dichlorisoprenaline* OR dihydroalprenolol* OR dilevalol* OR diprafenone* OR dramedilol* OR epanolol* OR ersentilide* OR esmolol* OR exaprolol* OR falintolol* OR flestolol* OR flusoxolol* OR hydroxybenzylpindolol* OR indenolol* OR iodopindolol* OR iprocrolol* OR isamoltane* OR isoxaprolol* OR labetalol* OR landiolol* OR levobunolol* OR levomoprolol* OR mepindolol* OR mercuderamide* OR metipranolol* OR metoprolol* OR moprolol* OR nadolol* OR nebivolol* OR nifenalol* OR oberadilol* OR oxprenolol* OR pafenolol* OR pamatolol* OR penbutolol* OR pindolol* OR practolol* OR primidolol* OR prizidilol* OR procinolol* OR pronetalol* OR propranolol* OR proxodolol* OR ridazolol* OR ritodrine* OR salcardolol* OR sandoz-204545* OR soquinolol* OR sotalol* OR spirendolol* OR talinolol* OR tazolol* OR tertatolol* OR tienoxolol* OR tilisolol* OR timolol* OR tolamolol* OR toliprolol* OR trasitensin* OR trepress* OR tribendilol* OR viskaldix* OR vortioxetine* OR xibenolol* OR zoleprodolol*):ab,ti) NOT ([Conference Abstract]/lim OR [Letter]/lim OR [Note]/lim OR [Editorial]/lim) AND [english]/lim
**Medline Ovid 302**
(exp Glioma/ OR Brain Neoplasms/ OR Central Nervous System Neoplasms/ OR (glioma* OR astrocytom* OR xanthoastrocytom* OR gliosarcom* OR oligodendrogliom* OR

Suppl1. Search syntax in different databases

ependymom* OR glioblastom* OR ((brain OR central nervous system OR cns OR intracerebral* OR cerebral* OR glial) ADJ3 (cancer* OR cavernoma* OR neoplas* OR tumo* OR malign*))).ab,ti.) AND (exp Adrenergic beta-Antagonists/ OR (((beta) ADJ3 (block* OR antagonis* OR sympatholytic* OR adrenolytic*)) OR adaprolol* OR afurolol* OR alprenolol* OR befunolol* OR bfe-55* OR bopindolol* OR bornaprolol* OR bromoacetylalprenololmenthane* OR bucindolol* OR bucumolol* OR bufetolol* OR bufuralol* OR bunitrolol* OR bunolol* OR bupranolol* OR butofilolol* OR carazolol* OR carpindolol* OR carteolol* OR carvedilol* OR cloranolol* OR deacetylmetipranolol* OR dexpropranolol* OR diacetolol* OR dichlorisoprenaline* OR dihydroalprenolol* OR dilevalol* OR diprafenone* OR ersentilide* OR exaprolol* OR falintolol* OR falintolol-oxalate* OR flestolol* OR hydroxybenzylpindolol* OR indenolol* OR iodopindolol* OR iprocrolol* OR isamoltane* OR isoxaprolol* OR labetalol* OR levobunolol* OR levomoprolol* OR mepindolol* OR mercuderamide* OR metipranolol* OR moprolol* OR nadolol* OR nifenalol* OR oberadilol* OR oxprenolol* OR pafenolol* OR pamatolol* OR penbutolol* OR pindolol* OR primidolol* OR prizidilol* OR procinolol* OR pronetalol* OR propranolol* OR proxodolol* OR ridazolol* OR soquinolol* OR sotalol* OR spirendolol* OR tazolol* OR tertatolol* OR tienoxolol* OR tilisolol* OR timolol* OR tolamolol* OR toliprolol* OR trasitensin* OR trepress* OR tribendilol* OR viskaldix* OR xibenolol* OR zoleprodolol* OR acebutolol* OR arotinolol* OR atenolol* OR bendacalol* OR bendacalol* OR betaxolol* OR bevantolol* OR bfe-55* OR bisoprolol* OR bopindolol* OR bornaprolol* OR bromoacetylalprenololmenthane* OR bucindolol* OR bucumolol* OR bufetolol* OR bufuralol* OR bunitrolol* OR bunolol* OR bupranolol* OR butofilolol* OR butoxamine* OR carazolol* OR carpindolol* OR carteolol* OR carvedilol* OR celiprolol* OR cetamolol* OR cicloprolol* OR cloranolol* OR cyanoiodopindolol* OR cyanopindolol* OR deacetylmetipranolol* OR dexpropranolol* OR diacetolol* OR dichlorisoprenaline* OR dihydroalprenolol* OR dilevalol* OR diprafenone* OR dramedilol* OR epanolol* OR ersentilide* OR esmolol* OR exaprolol* OR falintolol* OR flestolol* OR flusoxolol* OR hydroxybenzylpindolol* OR indenolol* OR iodopindolol* OR iprocrolol* OR isamoltane* OR isoxaprolol* OR labetalol* OR landiolol* OR levobunolol* OR levomoprolol* OR mepindolol* OR mercuderamide* OR metipranolol* OR metoprolol* OR moprolol* OR nadolol* OR nebivolol* OR nifenalol* OR oberadilol* OR oxprenolol* OR pafenolol* OR pamatolol* OR penbutolol* OR pindolol* OR practolol* OR primidolol* OR prizidilol* OR procinolol* OR pronetalol* OR propranolol* OR proxodolol* OR ridazolol* OR ritodrine* OR salcardolol* OR sandoz-204545* OR soquinolol* OR sotalol* OR spirendolol* OR talinolol* OR tazolol* OR tertatolol* OR tienoxolol* OR tilisolol* OR timolol* OR tolamolol* OR toliprolol* OR trasitensin* OR trepress* OR tribendilol* OR viskaldix* OR vortioxetine* OR xibenolol* OR zoleprodolol*).ab,ti.) NOT (letter* OR news OR comment* OR editorial* OR congres* OR abstract* OR book* OR chapter* OR dissertation abstract*).pt. AND english.la.
**Web of science 225**
TS=(((glioma* OR astrocytom* OR xanthoastrocytom* OR gliosarcom* OR oligodendrogliom* OR ependymom* OR glioblastom* OR ((brain OR "central nervous system" OR cns OR intracerebral* OR cerebral* OR glial) NEAR/2 (cancer* OR cavernoma* OR neoplas* OR tumo* OR malign*)))) AND ((((beta OR β) NEAR/2 (block* OR antagonis* OR sympatholytic* OR adrenolytic*)) OR adaprolol* OR afurolol* OR alprenolol* OR befunolol* OR bfe-55* OR bopindolol* OR bornaprolol* OR bromoacetylalprenololmenthane* OR bucindolol* OR bucumolol* OR bufetolol* OR

Suppl1. Search syntax in different databases

bufuralol* OR bunitrolol* OR bunolol* OR bupranolol* OR butofilolol* OR carazolol* OR carpindolol* OR carteolol* OR carvedilol* OR cloranolol* OR deacetylmetipranolol* OR dexpropranolol* OR diacetolol* OR dichlorisoprenaline* OR dihydroalprenolol* OR dilevalol* OR diprafenone* OR ersentilide* OR exaprolol* OR falintolol* OR falintolol-oxalate* OR flestolol* OR hydroxybenzylpindolol* OR indenolol* OR iodopindolol* OR iprocrolol* OR isamoltane* OR isoxaprolol* OR labetalol* OR levobunolol* OR levomoprolol* OR mepindolol* OR mercuderamide* OR metipranolol* OR moprolol* OR nadolol* OR nifenalol* OR oberadilol* OR oxprenolol* OR pafenolol* OR pamatolol* OR penbutolol* OR pindolol* OR primidolol* OR prizidilol* OR procinolol* OR pronetalol* OR propranolol* OR proxodolol* OR ridazolol* OR soquinolol* OR sotalol* OR spirendolol* OR tazolol* OR tertatolol* OR tienoxolol* OR tilisolol* OR timolol* OR tolamolol* OR toliprolol* OR trasitensin* OR trepress* OR tribendilol* OR viskaldix* OR xibenolol* OR zoleprodolol* OR acebutolol* OR arotinolol* OR atenolol* OR bendacalol* OR bendacalol* OR betaxolol* OR bevantolol* OR bfe-55* OR bisoprolol* OR bopindolol* OR bornaprolol* OR bromoacetylalprenololmenthane* OR bucindolol* OR bucumolol* OR bufetolol* OR bufuralol* OR bunitrolol* OR bunolol* OR bupranolol* OR butofilolol* OR butoxamine* OR carazolol* OR carpindolol* OR carteolol* OR carvedilol* OR celiprolol* OR cetamolol* OR cicloprolol* OR cloranolol* OR cyanoiodopindolol* OR cyanopindolol* OR deacetylmetipranolol* OR dexpropranolol* OR diacetolol* OR dichlorisoprenaline* OR dihydroalprenolol* OR dilevalol* OR diprafenone* OR dramedilol* OR epanolol* OR ersentilide* OR esmolol* OR exaprolol* OR falintolol* OR flestolol* OR flusoxolol* OR hydroxybenzylpindolol* OR indenolol* OR iodopindolol* OR iprocrolol* OR isamoltane* OR isoxaprolol* OR labetalol* OR landiolol* OR levobunolol* OR levomoprolol* OR mepindolol* OR mercuderamide* OR metipranolol* OR metoprolol* OR moprolol* OR nadolol* OR nebivolol* OR nifenalol* OR oberadilol* OR oxprenolol* OR pafenolol* OR pamatolol* OR penbutolol* OR pindolol* OR practolol* OR primidolol* OR prizidilol* OR procinolol* OR pronetalol* OR propranolol* OR proxodolol* OR ridazolol* OR ritodrine* OR salcardolol* OR sandoz-204545* OR soquinolol* OR sotalol* OR spirendolol* OR talinolol* OR tazolol* OR tertatolol* OR tienoxolol* OR tilisolol* OR timolol* OR tolamolol* OR toliprolol* OR trasitensin* OR trepress* OR tribendilol* OR viskaldix* OR vortioxetine* OR xibenolol* OR zoleprodolol*))) AND DT=(article) AND LA=(english)
**Cochrane CENTRAL 11**
((glioma* OR astrocytom* OR xanthoastrocytom* OR gliosarcom* OR oligodendrogliom* OR ependymom* OR glioblastom* OR ((brain OR 'central nervous system' OR cns OR intracerebral* OR cerebral* OR glial) NEAR/3 (cancer* OR cavernoma* OR neoplas* OR tumo* OR malign*))):ab,ti) AND ((((beta OR β) NEAR/3 (block* OR antagonis* OR sympatholytic* OR adrenolytic*)) OR adaprolol* OR afurolol* OR alprenolol* OR befunolol* OR bfe-55* OR bopindolol* OR bornaprolol* OR bromoacetylalprenololmenthane* OR bucindolol* OR bucumolol* OR bufetolol* OR bufuralol* OR bunitrolol* OR bunolol* OR bupranolol* OR butofilolol* OR carazolol* OR carpindolol* OR carteolol* OR carvedilol* OR cloranolol* OR deacetylmetipranolol* OR dexpropranolol* OR diacetolol* OR dichlorisoprenaline* OR dihydroalprenolol* OR dilevalol* OR diprafenone* OR ersentilide* OR exaprolol* OR falintolol* OR falintolol-oxalate* OR flestolol* OR hydroxybenzylpindolol* OR indenolol* OR iodopindolol* OR iprocrolol* OR isamoltane* OR isoxaprolol* OR labetalol* OR levobunolol* OR levomoprolol* OR mepindolol* OR mercuderamide* OR metipranolol* OR moprolol* OR nadolol* OR nifenalol* OR oberadilol* OR oxprenolol* OR pafenolol* OR pamatolol* OR

Suppl1. Search syntax in different databases

penbutolol* OR pindolol* OR primidolol* OR prizidilol* OR procinolol* OR pronetalol* OR propranolol* OR proxodolol* OR ridazolol* OR soquinolol* OR sotalol* OR spirendolol* OR tazolol* OR tertatolol* OR tienoxolol* OR tilisolol* OR timolol* OR tolamolol* OR toliprolol* OR trasitensin* OR trepress* OR tribendilol* OR viskaldix* OR xibenolol* OR zoleprodolol* OR acebutolol* OR arotinolol* OR atenolol* OR bendacalol* OR bendacalol* OR betaxolol* OR bevantolol* OR bfe-55* OR bisoprolol* OR bopindolol* OR bornaprolol* OR bromoacetylalprenololmenthane* OR bucindolol* OR bucumolol* OR bufetolol* OR bufuralol* OR bunitrolol* OR bunolol* OR bupranolol* OR butofilolol* OR butoxamine* OR carazolol* OR carpindolol* OR carteolol* OR carvedilol* OR celiprolol* OR cetamolol* OR cicloprolol* OR cloranolol* OR cyanoiodopindolol* OR cyanopindolol* OR deacetylmetipranolol* OR dexpropranolol* OR diacetolol* OR dichlorisoprenaline* OR dihydroalprenolol* OR dilevalol* OR diprafenone* OR dramedilol* OR epanolol* OR ersentilide* OR esmolol* OR exaprolol* OR falintolol* OR flestolol* OR flusoxolol* OR hydroxybenzylpindolol* OR indenolol* OR iodopindolol* OR iprocrolol* OR isamoltane* OR isoxaprolol* OR labetalol* OR landiolol* OR levobunolol* OR levomoprolol* OR mepindolol* OR mercuderamide* OR metipranolol* OR metoprolol* OR moprolol* OR nadolol* OR nebivolol* OR nifenalol* OR oberadilol* OR oxprenolol* OR pafenolol* OR pamatolol* OR penbutolol* OR pindolol* OR practolol* OR primidolol* OR prizidilol* OR procinolol* OR pronetalol* OR propranolol* OR proxodolol* OR ridazolol* OR ritodrine* OR salcardolol* OR sandoz-204545* OR soquinolol* OR sotalol* OR spirendolol* OR talinolol* OR tazolol* OR tertatolol* OR tienoxolol* OR tilisolol* OR timolol* OR tolamolol* OR toliprolol* OR trasitensin* OR trepress* OR tribendilol* OR viskaldix* OR vortioxetine* OR xibenolol* OR zoleprodolol*):ab,ti)
**Google scholar**
glioma|astrocytoma|xanthoastrocytoma|gliosarcoma|oligodendroglioma|ependymoma |glioblastoma "beta|β blockers|blocking|antagonists"
